# Supplementary material for: A New Species Nyctegretis seminigra sp. nov. (Pyralidae, Phycitinae) Revealed by Congruent Morphological and Mitogenomic Evidence
Source: Insects. 2025 Apr 14;16(4):413. doi: 10.3390/insects16040413 (PMC12027870; doi:10.3390/insects16040413)
Supplement: Supplementary file 1 [file insects-16-00413-s001.zip › Supplementary Mateirals/Supplementary Mateirals.docx]

**A new *Nyctegretis* species (Pyralidae,** **Phycitinae) from China, with first two complete mitochondrial genomes for the genus**

Linlin Yang ^a^, Yuxian Zhou^a b^, Yingdang Ren^a^ ^c*^

*a Institute of Plant Protection, Henan Academy of Agricultural Sciences, Zhengzhou 450002, China.*

*b Ningxia University, Yinchuan 750021, China.*

*c* Corresponding author. E-mail: renyd@126.com*

**Table S1.** Organization of the mitochondrial genome for *Nyctegretis* *seminigra* sp. nov. (Ns) and *N*. *triangulella* (Nt)

| **Gene** | **Position** | | **Size(bp)** | | **Start/ Stop codon** | | **Intergenetic nucleotides*** | | **Anticodon** | **Strand** |
| --- | --- | --- | --- | --- | --- | --- | --- | --- | --- | --- |
|  | Ns | Nt | Ns | Nt | Ns | Nt | Ns | Nt |  |  |
| *trnM* | 1-66 | 1-68 | 66 | 68 |  |  |  |  | CAT | J |
| *trnI* | 73-136 | 69-132 | 64 | 64 |  |  | 6 | 0 | GAT | J |
| *trnQ* | 134-202 | 130-198 | 69 | 69 |  |  | -3 | -3 | TTG | N |
| *nad2* | 247-1254 | 241-1254 | 1008 | 1014 | ATT/TAA | ATT/TAA | 44 | 42 |  | J |
| *trnW* | 1253-1319 | 1253-1321 | 67 | 69 |  |  | -2 | -2 | TCA | J |
| *trnC* | 1312-1374 | 1314-1377 | 63 | 64 |  |  | -8 | -8 | GCA | N |
| *trnY* | 1375-1439 | 1378-1443 | 65 | 66 |  |  | 0 | 0 | GTA | N |
| *cox1* | 1453-2983 | 1448-2978 | 1531 | 1531 | CGA/TAA | CGA/T | 13 | 4 |  | J |
| *trnL2* | 2984-3050 | 2979-3045 | 67 | 67 |  |  | 0 | 0 | TAA | J |
| *cox2* | 3051-3732 | 3046-3727 | 682 | 682 | ATT/T | ATT/T | 0 | 0 |  | J |
| *trnK* | 3733-3803 | 3728-3798 | 71 | 71 |  |  | 0 | 0 | CTT | J |
| *trnD* | 3804-3869 | 3799-3865 | 66 | 67 |  |  | 0 | 0 | GTC | J |
| *atp8* | 3870-4031 | 3866-4027 | 162 | 162 | ATT/TAA | ATT/TAA | 0 | 0 |  | J |
| *atp6* | 4025-4705 | 4021-4701 | 681 | 681 | ATG/TAA | ATG/TAA | -7 | -7 |  | J |
| *cox3* | 4705-5493 | 4701-5489 | 789 | 789 | ATG/TAA | ATG/TAA | -1 | -1 |  | J |
| *trnG* | 5496-5561 | 5492-5559 | 66 | 68 |  |  | 2 | 2 | TCC | J |
| *nad3* | 5562-5915 | 5560-5913 | 354 | 354 | ATT/TAG | ATT/TAG | 0 | 0 |  | J |
| *trnA* | 5921-5984 | 5912-5976 | 64 | 65 |  |  | 5 | -2 | TGC | J |
| *trnR* | 5985-6048 | 5981-6044 | 64 | 64 |  |  | 0 | 4 | TCG | J |
| *trnN* | 6046-6110 | 6042-6107 | 65 | 66 |  |  | -3 | -3 | GTT | J |
| *trnS* | 6114-6179 | 6112-6177 | 66 | 66 |  |  | 3 | 4 | GCT | J |
| *trnE* | 6180-6247 | 6178-6245 | 68 | 68 |  |  | 0 | 0 | TTC | J |
| *trnF* | 6248-6313 | 6254-6318 | 66 | 65 |  |  | 0 | 8 | GAA | N |
| *nad5* | 6314-8048 | 6319-8053 | 1735 | 1735 | ATT/T | ATT/T | 0 | -1 |  | N |
| *trnH* | 8049-8114 | 8054-8119 | 66 | 66 |  |  | 6 | 6 | GTG | N |
| *nad4* | 8114-9453 | 8119-9458 | 1340 | 1340 | ATG/TA | ATG/TA | -1 | -1 |  | N |
| *nad4L* | 9456-9743 | 9459-9746 | 288 | 288 | ATG/TAA | ATG/TAA | 2 | 0 |  | N |
| *trnT* | 9746-9810 | 9749-9812 | 65 | 64 |  |  | 2 | 2 | TGT | J |
| *trnP* | 9811-9875 | 9813-9879 | 65 | 67 |  |  | 0 | 0 | TGG | N |
| *nad6* | 9878-10411 | 9882-10415 | 534 | 534 | ATA/TAG | ATT/TAA | 2 | 2 |  | J |
| *cytb* | 10423-11571 | 10428-11576 | 1149 | 1149 | ATG/TAA | ATG/TAA | 11 | 12 |  | J |
| *trnS2* | 11575-11639 | 11581-11645 | 65 | 65 |  |  | 3 | 4 | TGA | J |
| *nad1* | 11660-12595 | 11663-12598 | 936 | 936 | ATA/TAG | ATA/TAA | 20 | 17 |  | N |
| *trnL1* | 12599-12668 | 12602-12668 | 70 | 67 |  |  | 3 | 3 | TAG | N |
| *rrnL* | 12666-14037 | 12666-14055 | 1372 | 1390 |  |  | -3 | -3 |  | N |
| *trnV* | 14038-14104 | 14044-14112 | 67 | 69 |  |  | 0 | -12 | TAC | N |
| *rrnS* | 14105-14878 | 14113-14893 | 774 | 781 |  |  | 0 | 0 |  | N |
| *A+T rich* | 14879-15198 | 14894-15205 | 320 | 312 |  |  | 0 | 0 |  |  |

*Positive numbers indicated intergenic length, negative numbers indicated overlapping length

**Table S2.** Nucleotide composition and skewness of mitochondrial gemome for *Nyctegretis seminigera* **sp. nov.** (Ns) and *N*. *triangulella* (Nt).

| **Feature** | **Length(bp)** | | **A+T%** | | **C+G %** | | **AT-skew** | | **GC-skew** | |
| --- | --- | --- | --- | --- | --- | --- | --- | --- | --- | --- |
|  | Ns | Nt | Ns | Nt | Ns | Nt | Ns | Nt | Ns | Nt |
| Whole genome | 15198 | 15205 | 79.2 | 80.4 | 20.8 | 19.6 | -0.051 | -0.054 | -0.221 | -0.194 |
| PCGs | 11184 | 11190 | 77.6 | 79 | 22.4 | 21 | -0.152 | -0.156 | 0.089 | 0.038 |
| tRNAs | 1455 | 1465 | 80.4 | 81.3 | 19.6 | 18.7 | 0.011 | 0.015 | 0.184 | 0.166 |
| rRNAs | 2146 | 2171 | 84.5 | 84.6 | 15.5 | 15.4 | 0.071 | 0.091 | 0.368 | 0.363 |
| A+T rich region | 320 | 312 | 93.1 | 93.3 | 6.9 | 6.7 | -0.074 | -0.071 | -0.275 | -0.612 |

**Table S3.** Codon usage in mitochondrial PCGs of *N. seminigera* **sp. nov.** (NS) and *N. triangulella* (Nt)

| Amino acid | Codon | N | | RSCU | | Amino acid | Codon | N | | RSCU | |
| --- | --- | --- | --- | --- | --- | --- | --- | --- | --- | --- | --- |
|  |  | Ns | Nt | Ns | Nt |  |  | Ns | Nt | Ns | Nt |
| Phe | UUU | 358 | 362 | 1.9 | 1.91 | Tyr | UAU | 168 | 182 | 1.81 | 1.94 |
|  | UUC | 19 | 18 | 0.1 | 0.09 |  | UAC | 18 | 6 | 0.19 | 0.06 |
| Leu2 | UUA | 431 | 430 | 4.86 | 5 | Ter* | UAA | 7 | 8 | 1.56 | 1.78 |
|  | UUG | 23 | 24 | 0.26 | 0.28 |  | UAG | 2 | 1 | 0.44 | 0.22 |
| Leu1 | CUU | 45 | 45 | 0.51 | 0.52 | His | CAU | 52 | 59 | 1.58 | 1.71 |
|  | CUC | 7 | 6 | 0.08 | 0.07 |  | CAC | 14 | 10 | 0.42 | 0.29 |
|  | CUA | 25 | 11 | 0.28 | 0.13 | Gln | CAA | 55 | 58 | 1.86 | 1.93 |
|  | CUG | 1 | 0 | 0.01 | 0 |  | CAG | 4 | 2 | 0.14 | 0.07 |
| Ile | AUU | 422 | 434 | 1.86 | 1.9 | Asn | AAU | 221 | 229 | 1.79 | 1.88 |
|  | AUC | 32 | 24 | 0.14 | 0.1 |  | AAC | 26 | 14 | 0.21 | 0.12 |
| Met | AUA | 270 | 288 | 1.84 | 1.83 | Lys | AAA | 98 | 101 | 1.83 | 1.82 |
|  | AUG | 24 | 26 | 0.16 | 0.17 |  | AAG | 9 | 10 | 0.17 | 0.18 |
| Val | GUU | 58 | 57 | 1.53 | 1.59 | Asp | GAU | 46 | 57 | 1.51 | 1.75 |
|  | GUC | 8 | 2 | 0.21 | 0.06 |  | GAC | 15 | 8 | 0.49 | 0.25 |
|  | GUA | 69 | 75 | 1.82 | 2.1 | Glu | GAA | 66 | 67 | 1.74 | 1.79 |
|  | GUG | 17 | 9 | 0.45 | 0.25 |  | GAG | 10 | 8 | 0.26 | 0.21 |
| Ser2 | UCU | 120 | 133 | 2.9 | 3.3 | Cys | UGU | 27 | 28 | 1.86 | 1.75 |
|  | UCC | 22 | 8 | 0.53 | 0.2 |  | UGC | 2 | 4 | 0.14 | 0.25 |
|  | UCA | 71 | 74 | 1.72 | 1.84 | Trp | UGA | 87 | 85 | 1.85 | 1.83 |
|  | UCG | 4 | 2 | 0.1 | 0.05 |  | UGG | 7 | 8 | 0.15 | 0.17 |
| Pro | CCU | 65 | 69 | 2.08 | 2.17 | Arg | CGU | 11 | 13 | 0.85 | 1 |
|  | CCC | 27 | 19 | 0.86 | 0.6 |  | CGC | 2 | 0 | 0.15 | 0 |
|  | CCA | 33 | 38 | 1.06 | 1.2 |  | CGA | 37 | 37 | 2.85 | 2.85 |
|  | CCG | 0 | 1 | 0 | 0.03 |  | CGG | 2 | 2 | 0.15 | 0.15 |
| Thr | ACU | 93 | 103 | 2.38 | 2.71 | Ser1 | AGU | 30 | 27 | 0.73 | 0.67 |
|  | ACC | 13 | 6 | 0.33 | 0.16 |  | AGC | 7 | 2 | 0.17 | 0.05 |
|  | ACA | 49 | 41 | 1.26 | 1.08 |  | AGA | 77 | 76 | 1.86 | 1.89 |
|  | ACG | 1 | 2 | 0.03 | 0.05 |  | AGG | 0 | 0 | 0 | 0 |
| Ala | GCU | 74 | 80 | 2.43 | 2.6 | Gly | GGU | 33 | 46 | 0.66 | 0.93 |
|  | GCC | 18 | 5 | 0.59 | 0.16 |  | GGC | 12 | 8 | 0.24 | 0.16 |
|  | GCA | 26 | 35 | 0.85 | 1.14 |  | GGA | 92 | 103 | 1.85 | 2.08 |
|  | GCG | 4 | 3 | 0.13 | 0.1 |  | GGG | 62 | 41 | 1.25 | 0.83 |

N, number of codons used in the total protein codon gene. RSCU, relative synonymous codon usage.


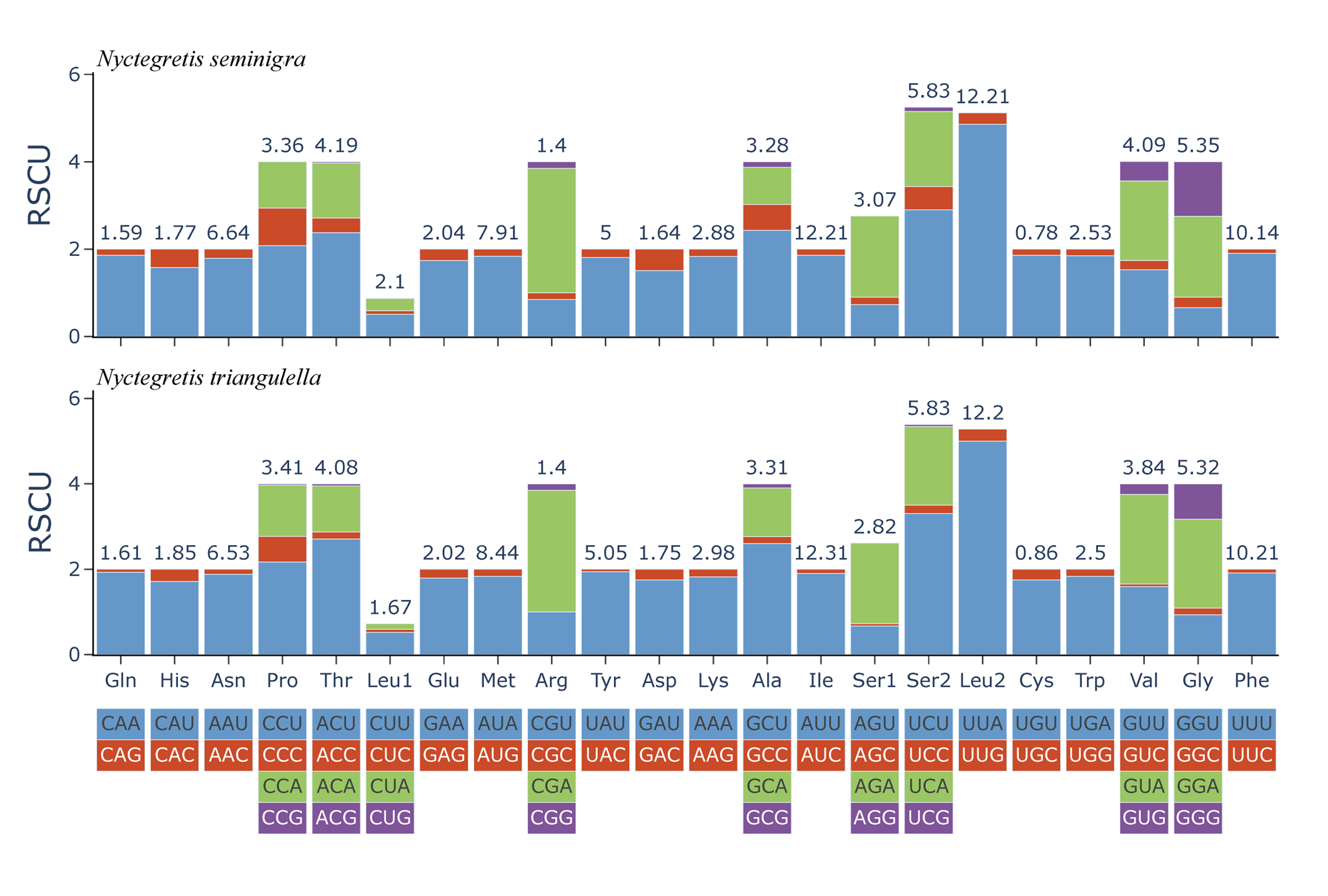


**Figure S1.** Amino acid frequency (values at the top of each bar) and relative synonymous codon usage of protein-coding genes in the mitogenomes of *Nyctegretis seminigra* sp. nov. and *N. triangulella*


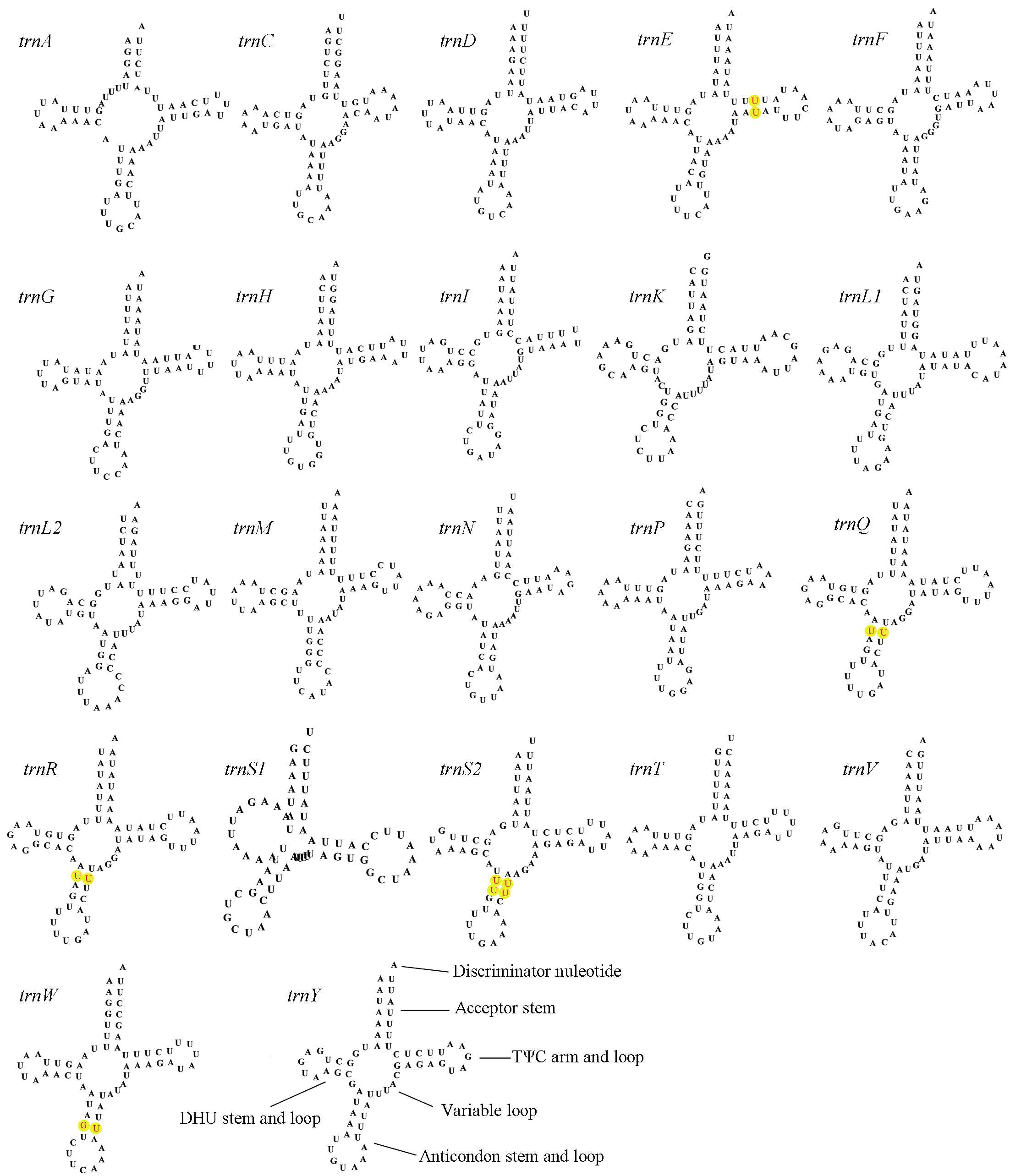


**Figure** **S2.** Predicted secondary structure of tRNAs in *Nyctegretis seminigera* s**p. nov.** mitogenome*.*

**Figure S3.** Predicted secondary structure of tRNAs in *Nyctegretis triangulella* mitogenome*.*
