# Supplementary figures and images for: A New Species Nyctegretis seminigra sp. nov. (Pyralidae, Phycitinae) Revealed by Congruent Morphological and Mitogenomic Evidence
Source: Insects. 2025 Apr 14;16(4):413. doi: 10.3390/insects16040413 (PMC12027870; doi:10.3390/insects16040413)

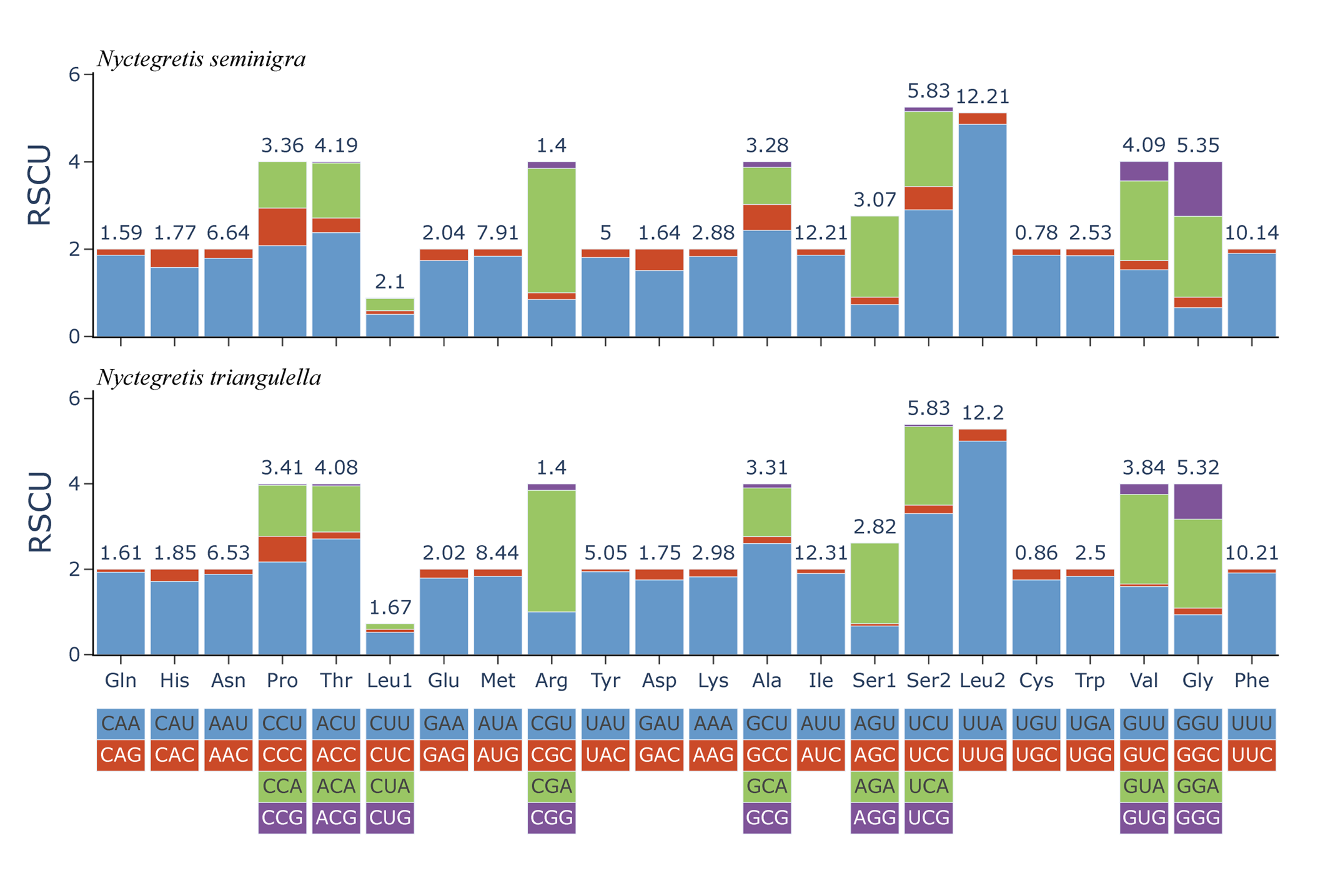

Supplement: Supplementary file 1 [file insects-16-00413-s001.zip › Supplementary Mateirals/Figure S1.tif]

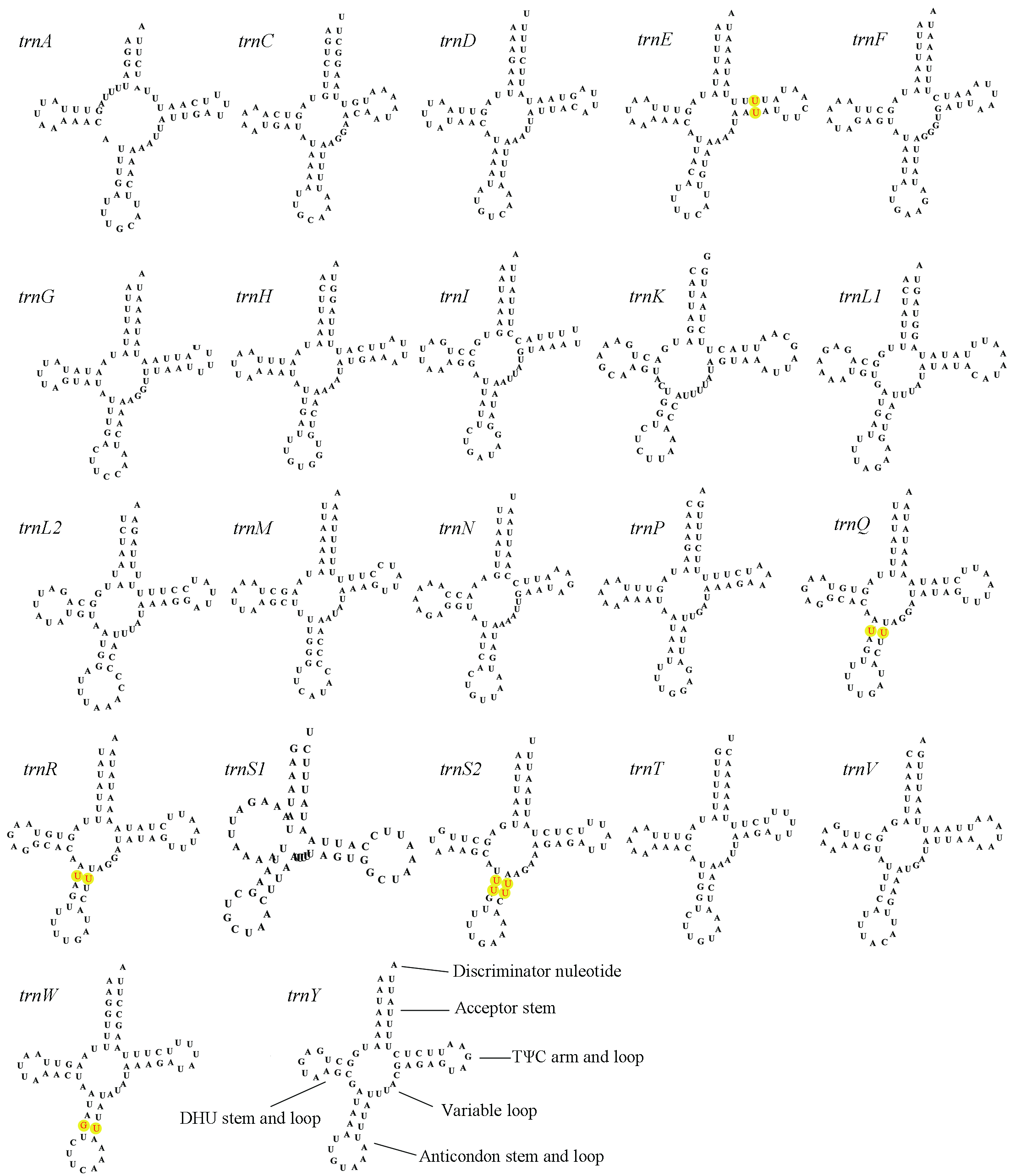

Supplement: Supplementary file 1 [file insects-16-00413-s001.zip › Supplementary Mateirals/Figure S2.tif]

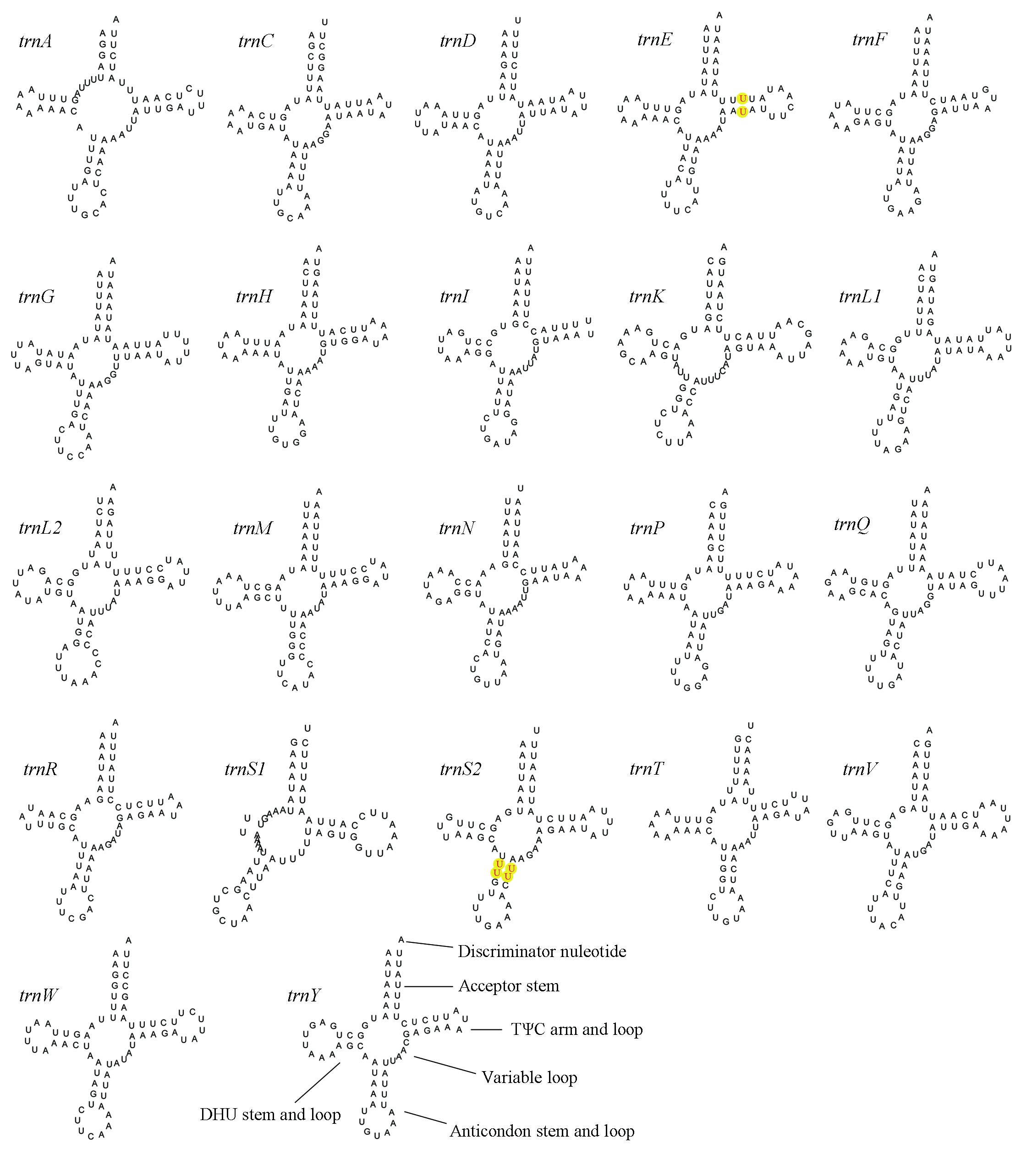

Supplement: Supplementary file 1 [file insects-16-00413-s001.zip › Supplementary Mateirals/Figure S3.tif]
